# A Transmembrane Domain GGxxG Motif in CD4 Contributes to Its Lck-Independent Function but Does Not Mediate CD4 Dimerization

**DOI:** 10.1371/journal.pone.0132333

**Published:** 2015-07-06

**Authors:** Heather L. Parrish, Caleb R. Glassman, Madeline M. Keenen, Neha R. Deshpande, Matthew P. Bronnimann, Michael S. Kuhns

**Affiliations:** 1 Department of Immunobiology, The University of Arizona College of Medicine, Tucson, Arizona, United States of America; 2 The Arizona Center on Aging, The University of Arizona College of Medicine, Tucson, Arizona, United States of America; 3 The BIO-5 Institute, The University of Arizona College of Medicine, Tucson, Arizona, United States of America; J. Heyrovsky Institute of Physical Chemistry, CZECH REPUBLIC

## Abstract

CD4 interactions with class II major histocompatibility complex (MHC) molecules are essential for CD4^+^ T cell development, activation, and effector functions. While its association with p56^lck^ (Lck), a Src kinase, is important for these functions CD4 also has an Lck-independent role in TCR signaling that is incompletely understood. Here, we identify a conserved GGxxG motif in the CD4 transmembrane domain that is related to the previously described GxxxG motifs of other proteins and predicted to form a flat glycine patch in a transmembrane helix. In other proteins, these patches have been reported to mediate dimerization of transmembrane domains. Here we show that introducing bulky side-chains into this patch (GGxxG to GVxxL) impairs the Lck-independent role of CD4 in T cell activation upon TCR engagement of agonist and weak agonist stimulation. However, using Forster’s Resonance Energy Transfer (FRET), we saw no evidence that these mutations decreased CD4 dimerization either in the unliganded state or upon engagement of pMHC concomitantly with the TCR. This suggests that the CD4 transmembrane domain is either mediating interactions with an unidentified partner, or mediating some other function such as membrane domain localization that is important for its role in T cell activation.

## Introduction

T cell development, activation, differentiation and effector functions are driven by signals generated by the recognition of peptides bound to major histocompatibility complex molecules (pMHC) on the surface of antigen presenting cells (APCs). The T cell receptor (TCR) is central to this process. It binds the composite surface formed by the peptide and MHC and relays information about the duration of these interactions to the immune receptor tyrosine-based activation motifs (ITAMs) within the intracellular domains of the associated CD3 signaling modules (CD3γε, δε, and ζζ,) [1, 2]. Information about peptide binding is then converted to chemical signals when the ITAMs are phosphorylated by the Src kinases p56^lck^ (Lck) or p59^fyn^ (Fyn) [3, 4]. The class II MHC co-receptor CD4 plays an important role in this process due to its association with Lck, but CD4 also makes an Lck-independent contribution to TCR signaling that is incompletely understood [5, 6].

The CD4 extracellular domain (ECD) has been structurally characterized to bind invariant portions of class II MHC via its D1 domain, while the intracellular domain (ICD) mediates interaction with Lck [7–14]. Previous studies have shown that a C-terminally mutated CD4, which lacks the cysteine clasp that mediates interactions with Lck, can nevertheless enhance TCR signaling *in vitro* [5]. C-terminally truncated CD4 can also rescue thymocyte development and selection in CD4-deficient mice. This provides further evidence for the multi-functional role of CD4 in TCR signaling that is isolated to the ECD and/or TMD [6].

The mechanisms underlying this Lck-independent function of CD4 are unclear. The affinity of the CD4 D1 domain for MHC is very weak, with estimates ranging from 200μM to 2mM, suggesting that it is unlikely to make a significant impact on the affinity of TCR interactions for pMHC [15]. This has led to the hypothesis that CD4 participates in homotypic or heterotypic interactions with additional cellular factor(s) via its ECD or TMD, or both, to mediate Lck-independent functions. We therefore searched for potential protein interaction motifs in CD4 that may contribute to this activity.

**Fig 1:**
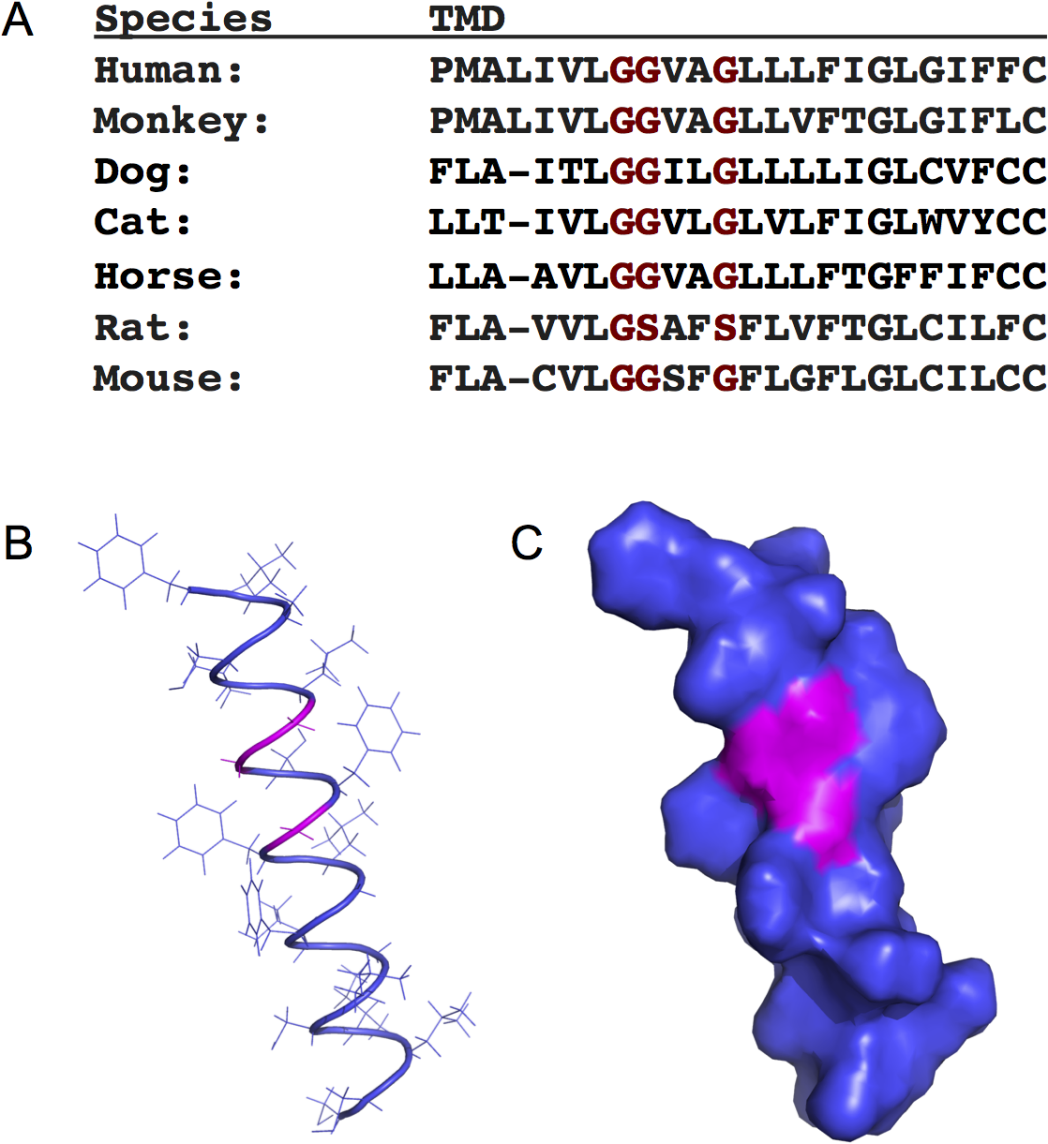
The CD4 TMD contains highly conserved glycine residues. (A) CD4 transmembrane domain (TMD) sequence alignment showing highly conserved glycine residues shaded red. (B) Ribbon diagram model or (C) space-filled model of the CD4 TMD with alanine and glycine residues of interest highlighted in magenta (generated with PyMol).

The GGxxG sequence in the CD4 transmembrane domain (TMD) represents one such motif (Fig 1A). The spacing of the glycines forms a side-chain free patch along one side of the TMD α-helix that allows tight packing of helices and possibly hydrogen bonding between peptide backbones of TMDs. Such patches have been reported to mediate homotypic or heterotypic interactions in a wide assortment of transmembrane proteins, including glycophorin A (GpA), scavenger receptor class B, type I (SRBI), and the human papilloma virus L2 transmembrane domain [16–18]. The conservation of this sequence in the CD4 TMD of a wide range of vertebrates suggested to us that it might play an important role in T cell activation (Fig 1A).

In this study, we investigated if the highly conserved GGxxG motif of the CD4 TMD contributes to T cell activation and CD4 dimerization. We report that the addition of bulky side chains to this surface (GVxxL), to introduce steric clashing at this putative dimerization interface, has no impact on CD4 expression but impairs its Lck-independent contribution to T cell activation in response to agonist and weak agonist TCR-pMHC interactions. Using Förster’s resonance energy transfer (FRET) we found that mutating the GGxxG motif does not inhibit homotypic CD4 interactions. This suggests that the CD4 TMD may mediate heterotypic interactions with other molecules involved in T cell activation or may serve an additional unknown function in the contribution of CD4 to TCR signaling.

## Materials and Methods

### Constructs

All constructs used in this study were built using the MSCV-based retroviral expression vectors pP2 (IRES-puromycin resistance) and pZ4 (IRES-zeocin resistance) [19, 20]. The proteins encoded by the constructs used in this study are described by amino acid (aa) number beginning at the start methionine (UniProt convention). The 5c.c7 TCR, which is specific to MCC 88–93 presented in I-E^k^, was used as the TCR for all experiments. Full-length CD3δ, ε, γ and ζ were encoded on a poly-cistronic construct as previously described [20, 21]. C-terminally truncated CD4, CD4T (amino acids: 1–421), was used to study the Lck-independent function of CD4 in order to restrict our study to the ECD or TMD of CD4 since this has previously been established to function in CD4 T cell development [6]. Site-directed mutagenesis was used to generate the CD4T^TMD^ (G403V/G406L) mutant. Mutations in the CD4 MHC class II binding domain (CD4T^Δbind^) were made by changing residues 68–73 (KGVLIR) to DGDSDS [13, 14]. For FRET experiments, the extracellular and transmembrane domains of wild-type or mutant CD4T (amino acids: 1–421), CD28 (amino acids: 1–179), and PD1 (amino acids: 1–199) were fused to mEGFP or mCherry via a short flexible linker (GSAAA). For expression of E^k^-MCC, E^k^-T102S and E^k^-HB in or M12 cells [22], the full I-E^k^ alpha subunit was expressed along with a full I-E^k^ beta subunit expressed as a fusion with the mouse hemoglobin d allele peptide (Hb 64–76), moth cytochrome c (MCC 88–103) peptide or T102S peptide at the N-terminus, via a short linker similarly to Kappler and colleagues [23].

### Cell lines and flow cytometry

M12 cells [22] expressing peptide:I-E^k^ and 58α^-^β^-^ cell lines [24] expressing the 5c.c7 TCR, full-length CD3 subunits and CD4 were generated as previously described [19, 25]. Multiple independent 58α^-^β^-^ cell lines were generated and tested in the functional and FRET assays to ensure that any phenotypes were as a consequence of the mutations and not cell culture divergence from the parental cells. Surface expression levels of CD4 (mAb clones GK1.5 e450, eBiosciences) and TCRβ VβC (mAb clone KJ25 PE, BD Bioscience) were assessed by low cytometry as indicated in Figures. Chinese Hamster Ovarian cells expressing I-E^k^ were previously described [26].

### Functional assays

For CHO I-E^k^ co-culture experiments 5 X 10^4^ 58α^-^β^-^ cells were co-cultured with 1 X 10^5^ CHO I-E^k^ cells and a titration of MCC 88–103 peptide in 96-well flat-bottom plates. Alternatively, 2.5 X 10^4^ 58α^-^β^-^ cells were co-cultured with 1 X 10^5^ M12 cells expressing I-E^k^ tethered to MCC, T102S or HB in 96-well round-bottom plates. For both assays supernatants were collected after 16 hours of co-culture at 37°C IL-2 was quantitated by ELISA. Anti-mouse IL-2 (clone JES6-1A12, Biolegend) was used as a capture antibody and biotin anti-mouse IL-2 (clone JES6-5H4, Biolegend) was used as the secondary antibody. Streptavidin-HRP and TMB substrate (Biolegend) were used for detection.

### Lipid bilayers

Bilayers were prepared similarly to previous studies [20, 27]. In brief, liposomes composed of 97.5 mol % 1-palmitoyl-2-oleoyl-*sn*-glycero-3-phosphocholine, 1 mol % 1,2-dioleoyl-*sn*-glycero-3-[(N-(5-amino-1-carboxypentyl)iminodiacetic acid)succinyl] (nickel salt), 1 mol % 1,2-dioleoyl-*sn*-glycero-3-phosphoethanolamine-N-(cap biotinyl) and 0.5 mol % 1,2-dioleoyl-*sn*-glycero-3-phosphoethanolamine-N-[methoxy(polyethylene glycol)-5000] (ammonium salt) were prepared by extrusion and injected onto a glass coverslip. Bilayer mobility was assed by photoablation recovery of streptavidin conjugated to PE. Each well received 0.05μg of MCC I-E^K^ and 0.08μg ICAM-1 to produce an agonist peptide density of approximately 60mol/micron^2^ [27].

### Soluble proteins for bilayers

Soluble class II pMHC monomers were generated with the baculovirus expression vector backbone pAcGP67A (Pharmingen). The full extracellular domain of I-E^k^ alpha (aa: 26–216) was expressed as a fusion with the acidic leucine zipper, a BirA acceptor peptide, and a 6X his tag similarly to the approach of Teyton and colleagues [28]. The I-E^k^ beta extracellular domain (31–225) was expressed as a fusion with the moth cytochrome c (MCC 88–103) peptide at the N-terminus, similarly to previous designs [23], and at the C-terminus with a basic leucine zipper and 6X his tag [28]. Purification was performed with Ni-NTA affinity resin (Qiagen) followed by size exclusion on an S200 (GE) via FPLC. ICAM-1 was produced as previously described [20].

### Microscopy

TIRFM was performed at 37°C, 5% CO_2_, and 50% relative humidity. Cells were adhered to the glass coverslip or lipid bilayers for 10 minutes and then imaged for 30–40 minutes following adhesion. TIRF images were acquired with a Zeiss fluorescent microscope using a 63X Zeiss TIRF objective coupled to a Zeiss motorized TIRF slider (NA 1.46). TIRFM was performed with a Laser Stack (3I) containing 50mW 488nm and 561nm solid-state lasers set at 20% power output. Photo ablation of mCherry was performed with a Vector high-speed point scanner at 75% 561nm laser output within a 25.8μm^2^ region of interest (412 pixels). All images were collected with 100-millisecond exposure at 500 millisecond intervals (Photometrics Evolve EMCCD; 100 intensification; 1 pixel = 0.25μm (H) x 0.25μm (V) at 63x).

### Image Analysis

Median mEGFP and mCherry intensity for the region of interest targeted for mCherry ablation were background subtracted using SlideBook6 (3I) and exported. Background subtracted mEGFP values for the time points immediately before and after photobleaching were used to calculate FRET_E_ = 1- (Q/DQ) where Q (quenched) is the mEGFP intensity prior to mCherry ablation and DQ (dequenched) is the mEGFP intensity following ablation as previously reported [29, 30]. mCherry ablation was calculated as the ratio of post to pre bleach mCherry intensity, Abl = mCh(postbleach)/mCh(prebleach). Cells with mCherry ablation below 0.125 were considered for analysis. Only cells with an mEGFP to mCherry ratio centered around 1 and ranging from 0.5 to 1.5 were considered for analysis. Further subset analysis was based on equivalent mean mEGFP and mCherry intensities.

### Statistical Analysis

All statistical analyses were performed with Prism 5.0 (GraphPad Software, Inc). The experiments involved normalized data or non-normally distributed cell populations. The Mann-Whitney t test or Kruskal-Wallis one-way analysis of variance (ANOVA) with a Dunn’s multiple comparison’s post-test using were performed where appropriate.

## Results

### Conserved glycines in the CD4 TMD contribute to T cell activation

To better understand the Lck-independent role of CD4 we looked for conserved amino acid motifs that are known to play a role in protein-protein interactions. We identified a GGxxG motif in the CD4 TMD that is absolutely conserved in humans, monkeys, dogs, cats, horses, rats and mice (Fig 1A). The conserved glycines in this motif are predicted to form a patch with limited steric hindrance along one interface of the CD4 transmembrane helix (Fig 1B and 1C). Modeling of the TMD revealed that Gly 403, while not part of a canonical GxxxG motif, also lies along the same face of the helix and would contribute to a larger glycine patch on one side of the CD4 TMD.

**Fig 2:**
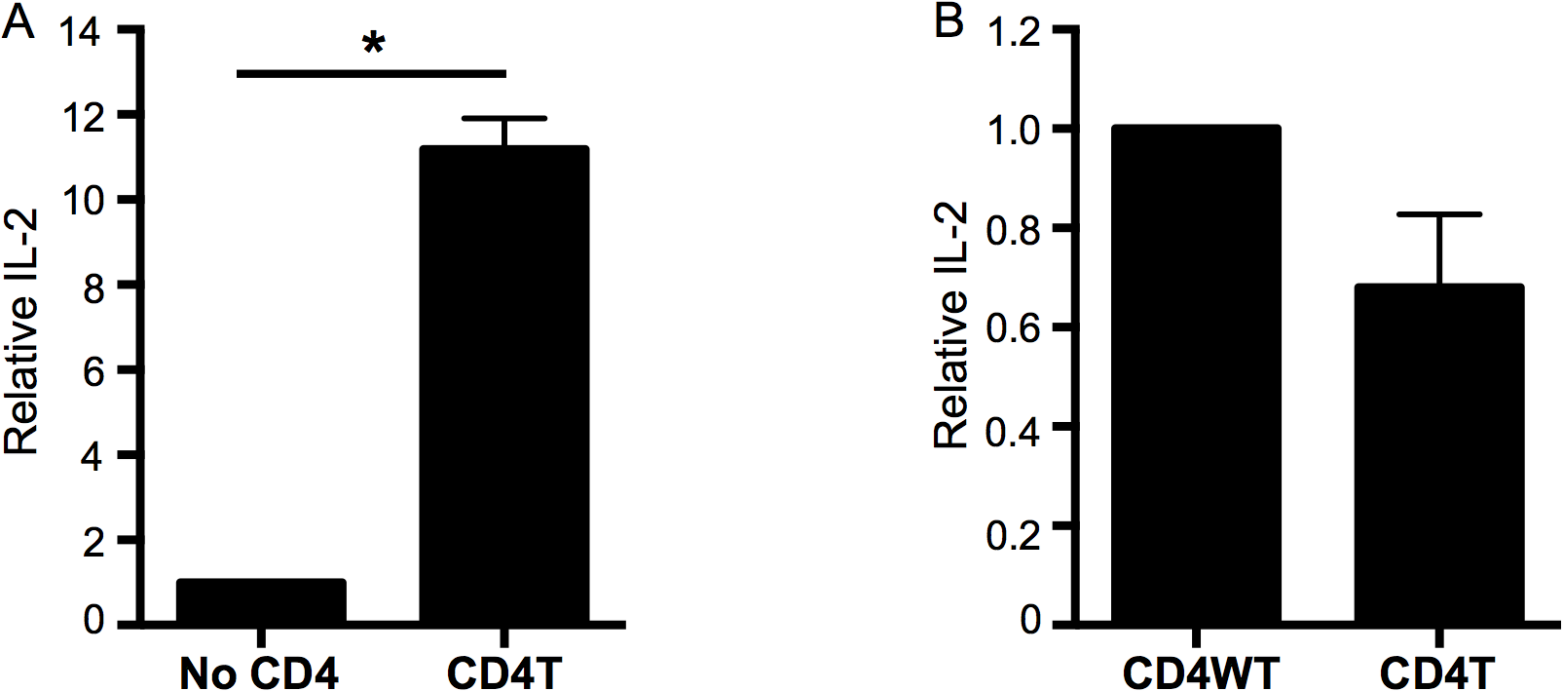
CD4T contributes to T cell activation. (A) 58α^-^β^-^ T cell hybridomas were retrovirally transduced with the 5c.c7 TCR and either CD4WT, CD4T or no CD4. IL-2 secretion from CD4T 58α^-^β^-^ T cell hybridomas after 16 hours of co-culture with MCC:I-E^k+^ M12 cells was normalized to the matched no CD4 controls within the same experiment to determine a relative IL-2 concentration. Bars represent mean values +/- SEM from four independently generated matched sets of cell lines. (B) IL-2 secretion after 16 hours of co-culture with MCC:I-E^k+^ M12 cells normalized to matched CD4WT controls within the same experiment to determine relative IL-2 concentration. Bars represent mean values +/- SEM from four independently generated matched sets of cell lines. (*p<0.05; Mann-Whitney).

Because GxxxG motifs and glycine patches are often involved in functionally important protein-protein interactions, we hypothesized that this GGxxG motif is important for CD4’s Lck-independent function in TCR signaling [16–18]. To test this hypothesis, we generated constructs encoding a wild type (CD4WT) or C-terminally truncated version of CD4 (CD4T). CD4T lacks the cysteine clasp that mediates interactions with Lck but has been reported to have Lck-independent function that can mediate CD4^+^ T cell development [6]. This molecule also lacks Cys 421 that has been reported to be palymitoylated, although mutation of this residue does not impact lipid raft localization of human CD4 [31, 32]. To verify that CD4T makes an Lck-independent contribution to T cell activation, we generated 58α^-^β^-^ T cell hybridomas expressing the 5c.c7 TCR, which recognizes a peptide from moth cytochrome c (MCC 88–93) in the context of I-E^k^, along with either CD4WT, CD4T, or no CD4. We observed a significant increase in IL-2 production from cells expressing CD4T relative to cells lacking CD4 expression when stimulated with M12 cells expressing I-E^k^ tethered to the agonist MCC peptide (Fig 2A). These data indicate that CD4T does contribute to T cell activation in an Lck-independent manner. This response trended lower than that of CD4WT cells, indicating that Lck-association with CD4 enhances T cell activation (Fig 2B).

**Fig 3:**
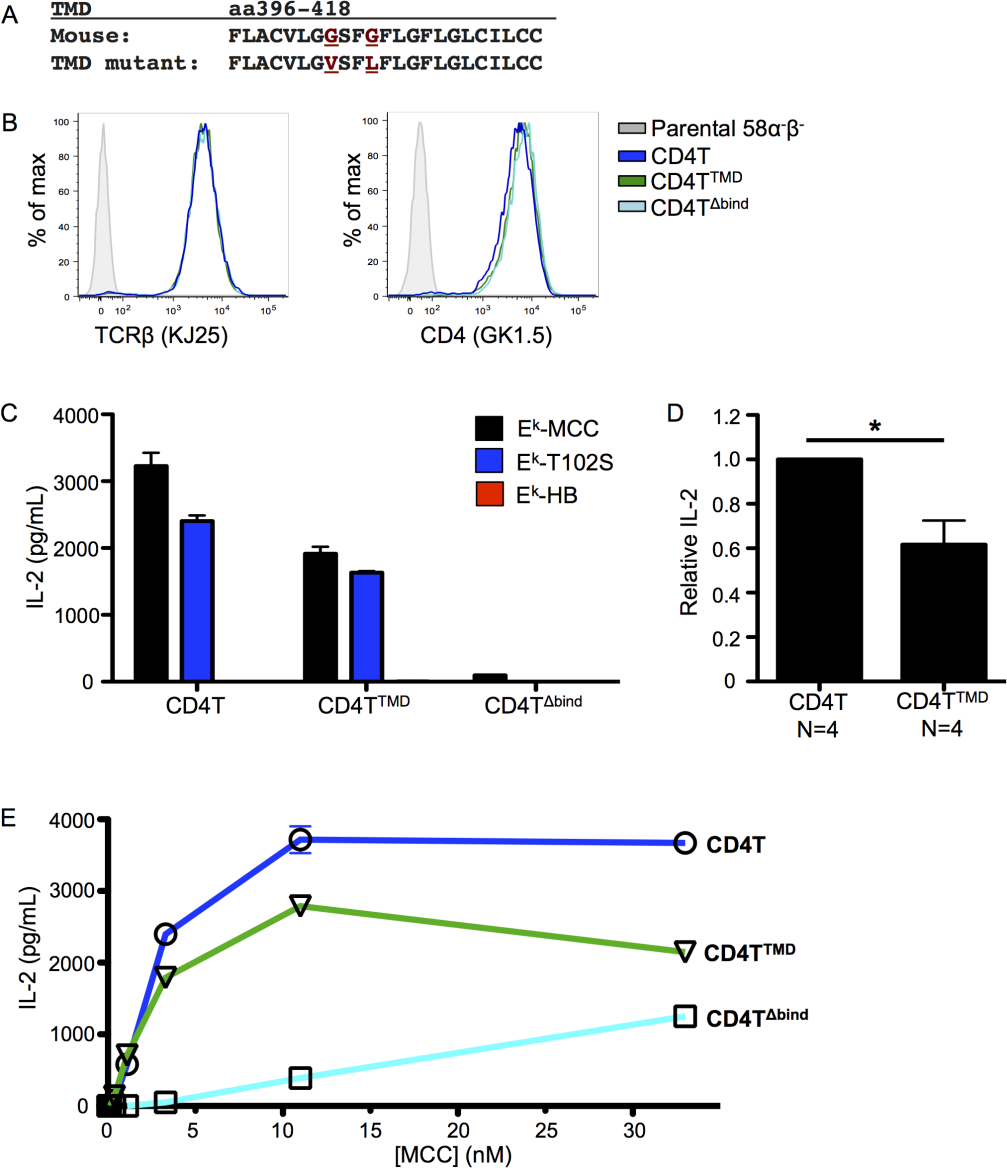
Mutating the CD4 transmembrane domain GGxxG motif impairs T cell activation. (A) Alignment of the wild-type CD4 TMD with the CD4 TMD mutant (G403V/G406L). Mutated residues are highlighted in red. Bulky side chains were introduced to disrupt the glycine patch. (B) 58α^-^β^-^ T cell hybridomas were retrovirally transduced with the 5c.c7 TCR and either CD4T, CD4T^TMD^ or CD4T^Δbind^, which is mutated in the region known to bind MHC class II. Surface expression of TCR and CD4 were assessed by flow cytometry as labeled. (C) IL-2 secretion from 58α^-^β^-^ T cell hybridomas after 16 hours of co-culture with M12 cells expressing agonist (MCC), weak agonist (T102S), or null (HB) peptide tethered to I-E^k^. Data are representative of four independent experiments with independently generated cell lines. (D) IL-2 secretion from four independently generated CD4T^TMD^ 58α^-^β^-^ T cell hybridomas after 16 hours of co-culture with MCC:I-E^k+^ M12 cells normalized to matched CD4T controls within the same experiment to determine relative IL-2 concentration. (E) IL-2 secretion from 58α^-^β^-^ T cell hybridomas after 16 hours of co-culture with Chinese hamster ovary (CHO) cells ectopically expressing I-E^k^ (CHO E^k^) pulsed with MCC peptide at the indicated concentrations. Data are representative of three independent experiments with independently generated cell lines. (*p<0.05; Mann-Whitney).

Having established a readout for the Lck-independent function of CD4, we generated a construct encoding a mutant of CD4T, referred to here as CD4T^TMD^, in which two of the glycines in the GGxxG motif were mutated to valine (G403V) or leucine (G406L) to introduce bulky side-chains at this surface (Fig 3A). As a negative control, we also generated a CD4T mutated in the region known to bind MHC class II (CD4T^Δbind^) [13, 14]. To test the function of these mutants we generated 58α^-^β^-^ T cell hybridomas expressing the 5c.c7 TCR along with CD4T, CD4T^TMD^, or CD4T^Δbind^. Cell surface expression of the TCR and CD4 were assessed by flow cytometry to ensure that the cell lines expressed equivalent levels of these proteins in order to assign any phenotype to the mutations rather than unmatched expression (Fig 3B).

First, we assessed the response of the CD4T^TMD^ mutant to stimuli of different affinity using the well-characterized altered peptide ligand (APL) for MCC, T102S, which has been previously characterized as a weak agonist [33, 34]. Here, we used M12 cells as APCs that expressed I-E^k^ tethered to MCC or T102S, or to a null peptide from mouse hemoglobin d allele peptide (Hb 64–76). In this assay, CD4T^TMD^ cells produced less IL-2 upon co-culture with APCs expressing E^k^-MCC or E^k^-T102S (Fig 3C). This was consistently observed with multiple independently generated sets of control and mutant cell lines (Fig 3D), demonstrating that the GGxxG motif plays a role in T cell activation.

We next assessed the response of the cell lines to stimulation with a titration of cognate antigen in the presence of Chinese Hamster Ovary (CHO) cells ectopically expressing I-E^k^ (CHO E^k^). Cells expressing CD4T^TMD^ produced less IL-2 in response to agonist pMHC stimulation than did those expressing CD4T (Fig 3E), thus providing additional evidence for a functional role of the GGxxG motif in T cell activation.

### The CD4 TMD GGxxG motif does not mediate dimerization at steady-state

CD4 has been reported to form homodimers upon crystallization due to contacts in the D4 domain of the ECD [35]. This interaction has been confirmed for human CD4 by mutagenesis coupled with both biochemical approaches and FRET, and has been shown to be functionally relevant [36, 37]. Additionally, other studies have shown involvement of the CD4 ICD in dimerization or multimerization [38, 39]. Since the glycine patches formed by GxxxG motifs often mediate homotypic interactions, we postulated that the CD4 GGxxG motif might constitute a portion of a dimerization interface.

FRET was employed to determine if the GGxxG motif contributes to CD4 dimerization. Since CD28 forms a disulfide-bonded homodimer, it served as a positive control, while PD1 is not reported to dimerize and thus served as a negative control. CD4WT, CD4T, and CD4T^TMD^ were fused to mEGFP or mCherry via a short flexible linker and expressed in 58α^-^β^-^ T cell hybridomas with the 5c.c7 TCR. C-terminally truncated forms of CD28 (CD28T) and PD1 (PD1T) were also fused to mEGFP or mCherry via the same short flexible linker and expressed with the 5c.c7 TCR in independent cell lines.

**Fig 4:**
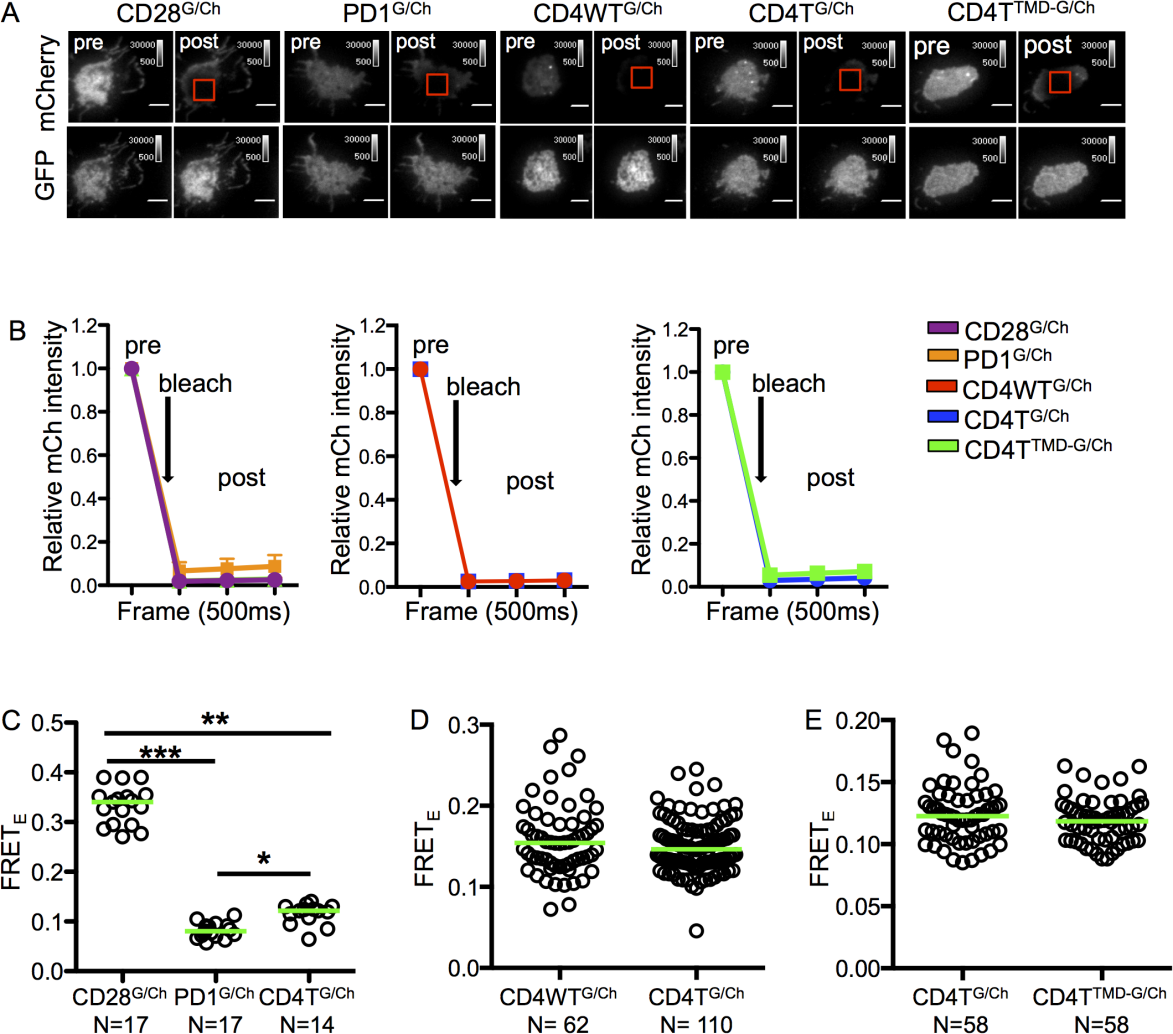
Mutating the CD4 TMD GGxxG motif does not impair dimerization at steady-state. (A) Grey-scale images of mCherry (top) and GFP (bottom) intensities pre and post mCherry ablation for representative cells. (B) Plot of relative mCherry values pre- and post-ablation for all cells analyzed. The average ablation of all populations was below 10% and did not differ significantly from each other. (C) FRET_E_ values for CD28^GFP/Ch^, PD1^GFP/Ch^ and CD4T^GFP/Ch^ cells. Representative of two experiments with independently generated cell lines. (D) FRET_E_ values for CD4WT^GFP/Ch^ vs. CD4T^GFP/Ch^ cells. Concatenated data from two independently generated cells lines is shown because the CD4WT^GFP/Ch^ cells had a broader range of donor and acceptor levels that limited the number of cells available for subset analysis (methods). (E) FRET_E_ values for CD4T^GFP/Ch^ vs. CD4T^TMD-GFP/Ch^ cells. Representative of two experiments with independently generated cell lines. Matched expression for analysis was based on median pre-bleach intensity. Dots represent single cells and green bars represent median values (*p<0.05, **p<0.001; ***p<0.0001; Mann-Whitney).

Live cells were imaged by total internal reflection fluorescence microscopy (TIRFM) after binding to glass coverslips [20]. This allowed us to limit our analysis to molecules at the cell surface without engaging the TCR or CD4 molecules. FRET efficiency values (FRET_E_) were quantified by measuring donor recovery after bleaching of the acceptor [29, 30]. In these experiments, ablation averaged greater than 90% for all analyzed populations and did not differ significantly from each other (Fig 4A and 4B). Furthermore, the analyzed cells were matched in their range of donor and acceptor intensities at the cell membrane as well as for their mean GFP and mCherry intensities. As expected, the positive control disulfide-bonded CD28T^G/Ch^ cells had a significantly higher FRET_E_ value compared to the negative control PD1T^G/Ch^ cells (Fig 4C). By comparison, the FRET_E_ values for the CD4T^G/Ch^ cells were significantly higher than the negative control cells, but significantly lower than those of the positive control cells. These data indicate that, while dimerization can occur, a low frequency of CD4 molecules dimerize or multimerize at equilibrium in these cells; thus, homotypic CD4 interactions are likely to be weak (Fig 4C). Of note, no significant difference was observed in the FRET_E_ value between lines expressing CD4WT^G/Ch^ or CD4T^G/Ch^ (Fig 4D). We then compared CD4T^TMD-G/Ch^ cells to CD4T^G/Ch^ cells and did not observe decreased FRET_E_ values, implying that G403 and G406 are not involved in stabilizing CD4 dimerization at steady-state (Fig 4E).

### The CD4 TMD GGxxG motif does not mediate dimerization upon TCR engagement

**Fig 5:**
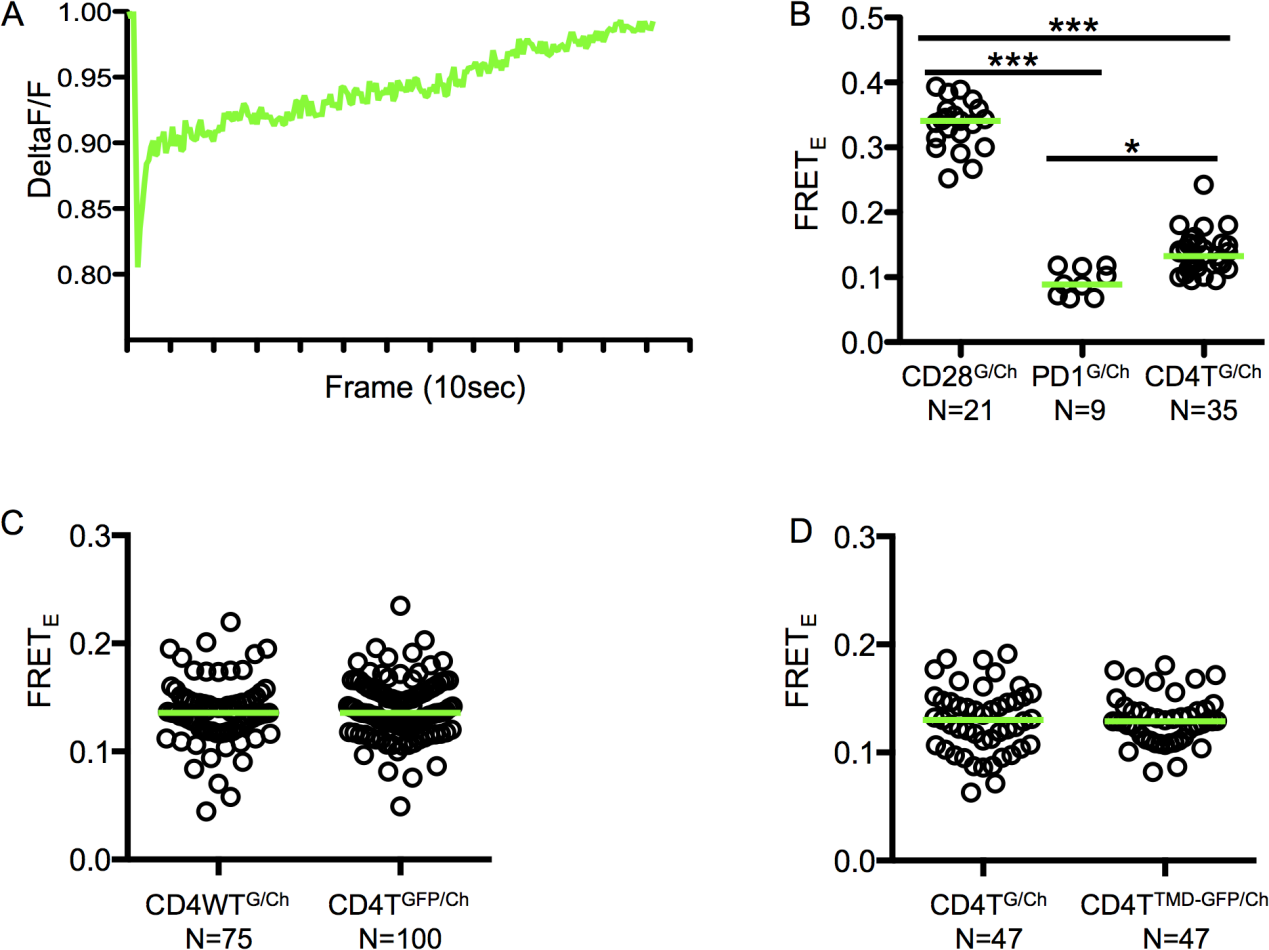
Mutating the CD4 TMD GGxxG motif does not impair dimerization upon TCR engagement. (A) Mobility of lipid bilayers was assessed by measuring recovery of streptavidin-PE molecules into a bleached region of the lipid bilayer and normalized to a reference region that was not bleached. (B) FRET_E_ values for CD28^GFP/Ch^, PD1^GFP/Ch^ and CD4T^GFP/Ch^ cells imaged by TIRFM on mobile bilayers containing agonist pMHC (MCC-E^k^). Data are representative of those obtained with two independently generated cell lines. (C) FRET_E_ values for CD4WT^GFP/Ch^ vs. CD4T^GFP/Ch^ cells imaged by TIRFM on mobile bilayers containing agonist pMHC (MCC-E^k^). Data is concatenated from two independently generated cell lines as in Fig 4D. (D) FRET_E_ values for CD4T^GFP/Ch^ vs. CD4T^TMD-GFP/Ch^ cells imaged by TIRFM on mobile bilayers containing agonist pMHC (MCC-E^k^). Data are representative of those obtained with two independently generated cell lines. Analysis was performed as for Fig 4. Dots represent single cells and green bars represent median values (*p<0.05, **p<0.001; ***p<0.0001; Mann-Whitney).

Previous studies using human CD4 have shown that CD4 dimerization increased upon TCR engagement [37]. Because we observed no decrease in FRET_E_ values of CD4T^TMD-G/Ch^ cells at steady-state compared to CD4T^G/Ch^, but observed a functional decrease in T cell activation with CD4T^TMD^ cells compared to CD4T cells, we hypothesized that the TMD glycine patch may contribute to the increase in CD4 dimerization that has been reported when both CD4 and the TCR engage pMHC. To test this hypothesis we imaged live cells using TIRFM on mobile lipid bilayers containing agonist (MCC-E^k^) pMHC. To ensure bilayer mobility, lipids were spiked with a lipid with a biotinylated head group. Streptavidin-PE was added to the bilayers and an area was bleached. Emission of PE was measured before and after bleaching to measure the ability of the bleached area to fill in with new PE molecules (Fig 5A). Similar to what we observed in cells with unengaged TCRs, the positive control CD28T^G/Ch^ cells had significantly higher FRET_E_ values than the negative control PD1T^G/Ch^ cells. CD4T^G/Ch^ cells had FRET_E_ values lower than CD28T^G/Ch^ cells but significantly higher than PD1T^G/Ch^ cells (Fig 5B). No difference in FRET_E_ was observed between cells expressing CD4WT^G/Ch^ and CD4T^G/Ch^ (Fig 5C). Likewise, CD4T^TMD-G/Ch^ cells had equivalent FRET_E_ values compared to CD4T^G/Ch^ cells, indicating that even after TCR engagement the CD4 TMD glycine patch is not involved in CD4 dimerization (Fig 5D). Of note, these hybridomas do not form classic immunological synapses on bilayers with a central cluster of TCR-CD3 complexes and we do not observe obvious segregation of TCR-CD3 and CD4 molecules after extended incubation on the bilayers, as has been reported for T cell lines or *ex vivo* T cell blasts [40, 41]. Since we did not observe any obvious differences in FRET between the various CD4 lines tested we think it unlikely that differences in segregation have impacted our analysis or interpretation.

## Discussion

The CD4 co-receptor for class II MHC plays an important role in TCR signaling. This is due, in part, to recruiting the Src kinase Lck to the TCR-CD3 complex [10–12]. However, there is also a well established but poorly defined role of CD4 in TCR signaling that is independent of Lck recruitment [5, 6]. To begin to understand the Lck-independent role of CD4 we first sought to identify conserved protein motifs that may contribute to this activity. In this study, we identified a highly conserved GGxxG motif in the CD4 transmembrane domain and show evidence that this glycine patch has an important functional role in T cell activation. Mutating two glycines in the CD4 TMD glycine patch reduced T cell hybridoma activation in response to a titration of agonist peptide as well as to weak agonist stimulation, indicating these residues are important for the Lck-independent function of CD4.

Because GxxxG motifs often mediate dimerization and multimerization of transmembrane domains and CD4 has been reported to form weak homodimers, we tested whether the CD4 TMD glycine patch was involved in such interactions [16–18, 38, 39]. Using FRET we confirmed that CD4 does form weak dimers, but our CD4 mutants, in which the TMD glycine patch was disrupted, did not have significantly lower FRET values than wild-type controls. This suggests that the glycine patch may be involved in heterotypic interactions with another protein or it may mediate some other function in TCR signaling.

The literature suggests several potential heterotypic interactions. CD4 has been suggested to interact directly with the TCR-CD3 complex, and it is possible that the CD4 TMD glycine patch contributes to this interaction [2, 42]. For example, the mouse CD3γ subunit TMD contains a GxxxA motif that is closely related to the GxxxG motif and may allow tight packing of α-helices and potential hydrogen bonding between peptide backbones of two or more helices. In addition, it has been reported that an AxxxG motif in the HIV gp41 fusion peptide interacts with a conserved motif in the TMD of the TCR alpha subunit to disrupt TCR signaling [43], so CD4 may also interact with this TCR motif. Furthermore, because CD4 is the receptor for HIV cellular entry [44] the AxxxG motif in HIV gp41 fusion peptide may somehow interact with the CD4 GGxxG motif and aid in HIV fusion with host cells. Alternatively, the GGxxG motif may facilitate interactions between CD4 and an unidentified protein, in which case CD4 may serve as a bridge to either stabilize interactions with the TCR-CD3 complex or help recruit CD4 in close proximity to the TCR-CD3 complex. Another possibility is that this motif may not specifically mediate interactions with another protein, but may be important for allowing another protein to approach CD4 and assume a close proximity while interacting specifically via a different domain. In such a case, our mutations may prevent this close association. These possibilities will be explored in future studies.

Finally, the GGxxG motif may contribute to CD4-assisted TCR signaling via some mechanism that does not involve protein-protein interactions. For instance, glycines are very flexible and may introduce conformational flexibility intoα-helices [45]. Thus, the glycines in the CD4 TMD may contribute flexibility into the CD4 TMD that assist in the Lck-independent contribution of CD4 signaling in some unknown way. Biochemical data have shown that, prior to TCR engagement, CD4 and the TCR inhabit distinct membrane domains and this may be important for their function [46]. There are several proteins whose TMD is required for their localization within distinct membrane domains. For example, CD40 and CD44 are both targeted to detergent resistant microdomains via their transmembrane domain [47–49]. Additionally, a GxxxG motif in mouse MHC class II I-A^k^ TMD has been shown to localize it to lipid rafts [50]. It is therefore possible that the TMD of CD4 plays an important role in its proper membrane localization. Heterotypic interactions or conformational flexibility mediated by the GGxxG motif could help facilitate this process.

In closing, the data presented here show that the CD4 TMD GGxxG motif contributes to the Lck-independent role of CD4 in T cell activation. Interestingly, this contribution is not due to dimerization of CD4 along this motif, indicating that it plays a distinct role in CD4-assisted T cell activation. Future work will be aimed at determining whether the CD4 GGxxG motif is important for mediating direct interactions with the TCR-CD3 complex to aid in signaling.
