# Correction: A Transmembrane Domain GGxxG Motif in CD4 Contributes to Its Lck-Independent Function but Does Not Mediate CD4 Dimerization

**DOI:** 10.1371/journal.pone.0150876

**Published:** 2016-03-01

**Authors:** Heather L. Parrish, Caleb R. Glassman, Madeline M. Keenen, Neha R. Deshpande, Matthew P. Bronnimann, Michael S. Kuhns

There is an error in the penultimate sentence of the “Constructs” section of the Materials and Methods. The correct sentence is: For FRET experiments, the extracellular and transmembrane domains of wild-type or mutant CD4T (amino acids: 1–421), CD28 (amino acids: 1–179), and PD1 (amino acids: 1–199) were fused to mEGFP or mCherry via a short flexible linker (AAAG).
